# Hospital at home (virtual wards): developing a logic model and dark logic model

**DOI:** 10.1186/s12913-025-12872-w

**Published:** 2025-05-17

**Authors:** Faiza Yahya, Matthew Cooper, Hamde Nazar

**Affiliations:** NIHR Newcastle Patient Safety Research Collaboration, Newcastle University, School of Pharmacy, Newcastle-upon-Tyne, NE1 7RU UK

**Keywords:** Hospital at home, Virtual wards, Healthcare system, Healthcare delivery, Integrated care, Patient safety, Logic model, Dark logic

## Abstract

**Background:**

Hospital at home (HaH), also referred to as virtual wards in the UK, enable patients to get hospital-level care at home with the use of digital technology, multidisciplinary teams, and remote monitoring. Despite recent evidence and rapid implementation, many questions around safe implementation and wider implications remain unanswered. Developing a logic model and dark logic model aimed to illustrate the recent evidence base with input from key informants and conceptualise the research focus for further work.

**Methods:**

Triangulation of three workstreams for comprehensiveness and credibility, involved (1) document analysis using publicly available documents, and non-published documents (including grey literature) provided by key stakeholders or virtual ward forums, (2) key informant interviews with a variety of expertise involved in the planning, implementation, or delivery of HaH, and (3) a focus group, to reach consensus on the final refined logic models. These were analysed using content analysis using an inductive and deductive approach to refine the logic models after each workstream.

**Results:**

A draft logic model was developed from document analysis describing key components of the logic model and dark logic models. Interviews with 12 participants helped refine the logic models with a subsequent focus group for consensus. The key themes for sustainability were securing clinical ‘buy-in’, effective communication, potential workforce re-modelling and optimising operational capabilities. Concerns and challenges were raised such as continuous funding, inadequate shared systems and duplication.

**Conclusion:**

These logic models provide a clear visual representation of intended (logic) and unintended outcomes (dark logic) of HaH (virtual wards) in England, and factors contributing to them. They can support prioritising future research or program planning and evaluation. Future research should explore strategies to deliver this personalised holistic care safely and effectively whilst maximising potential of resources like digital technology and understanding it’s impact on patients and equity.

**Supplementary Information:**

The online version contains supplementary material available at 10.1186/s12913-025-12872-w.

## Key messages

⇒ What is already known on this topic- Hospital at home models have been implemented at pace and variably across the nation and internationally. Evidence is inconsistent and many research gaps exist which impact on determination of the sustainability of services, patient safety and quality of care.

⇒ What this study adds- This study allows us to draw on current evidence with key stakeholder involvement and to visualise key components of HaH for achieving intended outcomes (e.g., success factors) but also potentially unintended outcomes (e.g., adverse effects/consequences).

⇒ How this study might affect research practice or policy -The results of the study allow us to focus on areas for quality improvement by exploring where underlying risks and challenges lie and prioritise these for future research and programme planning.

**Supplementary Information:**

The online version contains supplementary material available at 10.1186/s12913-025-12872-w.

## Background

Implementation of Hospital at Home (HaH), also referred to as virtual wards in the United Kingdom (UK), are rapidly increasing within the National Health Service (NHS) [1]. These innovative models enable patients to receive hospital-level care conveniently at home with the use of digital technology, multidisciplinary teams, and remote monitoring [2]. 

In the UK, virtual wards were first employed in the early 2000’s [3]. However, the terms and context have evolved over the years [2, 4–8]. NHS England (NHSE) defines HaH as:

*“Virtual wards (also known as hospital at home) allow patients to get hospital-level care at home safely and in familiar surroundings*,* helping speed up their recovery while freeing up hospital beds for patients that need them most. Just as in hospital*,* people on a virtual ward are cared for by a multidisciplinary team [MDT] who can provide a range of tests and treatments.”* [3]

The pressures post- COVID-19 pandemic have meant that virtual wards have formed an ambitious part of the NHSE urgent and emergency care recovery plan to scale-up with a goal of treating up to 50,000 patients a month [6]. This additional drive for rapid implementation to support healthcare recovery has led to variations nationally, with some confusion as to the correct terminology to explain what a ‘virtual ward’ entails, or the degree of technology used for remote monitoring [7, 8].

Rapid evidence syntheses [7] and realist reviews [9, 10] have sought to evaluate virtual wards, hospital at home (HaH) and remote monitoring (which uses technology but may not be restricted to patients requiring hospital level care). They have identified that variations in definitions and models of care exist. This heterogeneity, low quality, and deficient consistent evidence of effectiveness means the representability and relevance to the NHS healthcare system in England is unclear. Whilst it has been highlighted that a combination of components such as good co-ordination, appropriate MDTs, information sharing processes, proactive planning are important for effectiveness [9], there remain multiple research gaps such as around their implementation and identifying impacts on patients and their caregivers [7, 9]. 

Therefore, there is a clear need for mapping the evidence of HaH in England. One way of doing this is by developing logic models to represent the intended outcomes and impact but also a dark logic model to highlight risks and unintended outcomes to support areas for prioritisation of research or programme planning.

Logic models have been widely used in the health and social care sector as a helpful alternative tool for synthesising diverse evidence or mapping and evaluating complex interventions when there may be multiple inputs, outputs, and outcomes [11–15]. Other types of semi-structured logic models have also been adopted to improve implementation of evidence-based interventions in healthcare systems, aiming to provide a clear “roadmap” to understand connections between components to achieve desired outcomes [16]. While logic models usually only focus on the hypothesised intended benefit, contrastingly, ‘dark logic models’ anticipate the most likely unintended negative consequences, potential adverse effects and associated mechanisms that can lead to harm [17, 18].

This study sought to collate the evidence with stakeholder input to develop a logic model and dark logic model to visually depict the complexity of virtual wards and explore the research gaps to prioritise further work. Whilst much of the literature in England refers to virtual wards, this paper will be using the term HaH (in line with international guidance) [8, 19, 20] to cover both virtual wards and HaH.

This study aimed to answer the following questions:

1. What are the main intended outcomes and impact from HaH models and what inputs, activities and outputs are needed to achieve these?
2. What are the possible unintended consequences in HaH and what inputs, activities and outputs may potentially lead to these?
3. What are the research areas in HaH care that should be prioritised based on the logic models?

## Methods

This study followed the general principles described by WK Kellogg Foundation: Logic model development guide [11] and involved triangulation of data from three workstreams for comprehensiveness and credibility (document analysis, key informant interviews and a focus group). Due to the variability of HaH and the uniqueness of the UK healthcare system compared to other countries [21], it was decided that this study would focus predominantly on the UK literature. This study was intended to support future work in the UK healthcare system to align with the NHS ambitions of increasing capacity in virtual wards in England [1]. 

### Document analysis

#### Inclusion criteria and search strategy

Scoping searches identified several policy documents and national guidance on HaH, including rapid evaluations and recommendations. Documents published within the past five years were sought so that the information was relevant and current. Documents had to explicitly refer to virtual wards/HaH in the UK and not just HaH, remote monitoring or telemonitoring interventions independently. Citations included in the relevant evidence syntheses and policies were reviewed and involved a method of snowball searching. A search was conducted for grey literature on official NHS websites, Google, Social Care Institute for Excellence, and websites of professional membership bodies (such as the Royal Pharmaceutical Society and Royal College of Physicians). A cloud-based virtual ward collaboration platform (FutureNHS) was also searched for rapid evaluations commissioned by NHSE. Reports, commentaries and blogs on virtual ward use and implementation were also included. Documents were also signposted or provided by key informants.

#### Data extraction and analysis

Documents were collated and reviewed for suitability and followed the READ approach for document analysis [22]. Key concepts in the data were systematically mapped to the basic headings of a logic model (Inputs, Activities, Outputs, Outcomes, Impact) [8]. Following data extraction, data analysis and interpretation involved a combined inductive and deductive approach to inform preliminary draft logic models. Discussions took place between reviewers (FY, MC, HN) to gain consensus on interpretation of the data, the level of data required, and the suitability of the data within the components of the logic model considering the hypothesised causal links between categories [23]. The process was iterative, until consensus was gained among reviewers, on a distilled preliminary logic model.

The dark logic model was developed through a combination of identified concepts and concerns in the literature and theorised adverse effects or harms identified from the scrutiny and interpretation of the logic model. This methodology formed the foundations for discussions and triangulation with interviews and a focus group in later workstreams [24]. 

### Key informant interviews

Key informant interviews were conducted with diverse professionals involved in the design, implementation (management and organisation), and delivery of virtual wards. Interviews aimed to validate and build on the findings from the document analysis.

#### Sampling and recruitment

Stakeholder mapping was carried out by the researchers (FY, HN) identifying key contacts known to be involved in virtual wards. The relevant NHS Research and Development departments were contacted via email to seek permission, where applicable. An introductory email was sent by the primary researcher to key contacts (purposive sampling) and further suitable contacts were recruited through snowball sampling.

A participant information sheet was provided to participants before fully informed consent was obtained. A semi-structured interview guide (informed by the logic models) was used to guide discussions (Appendix 6). Sample size was guided by information power of the key informants to ensure richness and data sufficiency for refining the logic models [25, 26].

#### Analysis

The interviews were recorded and transcribed verbatim via MS Teams to prepare for content analysis [27, 28]. This systematic process consisting of three phases; preparing, organising and reporting the data [27] involved a combination of inductive and deductive analysis [28]. The analysis aimed to reach a stage where the concepts fit comfortably within the categories of the conceptual logic models.

Deductive content analysis was used to test the categories that were previously developed in the document analysis. This process allowed for re-wording and re-arrangement of some of the concepts. It also allowed for identification of new concepts highlighted as important by stakeholders and were not picked up by document analysis. This involved inductive content analysis. In this process, the data was organised by open coding, creating categories and abstraction [27]. Open coding was used to form concepts as described by Williams and Moser [29] and then creating categories, also known as axial coding [29]. Finally, further familiarisation of the data continued allowing abstraction to take place. This involved generating sub-categories and categories to condense the information and continued as far as reasonable.

During this process, the logic models were reviewed and adapted iteratively after every third transcript was analysed until no new amendments could be made and the researchers felt that the data portrayed a comprehensive overview of the information required.

### Focus group

The purpose of the focus group was a consensus development exercise to finalise and validate the refined logic models developed from previous workstreams.

#### Sampling and recruitment

Individuals who participated in the key informant interviews were sent invitations for the online focus group. A date and time was arranged to align with the availability of consenting participants. Participants were also sent the refined logic models via email in advance of the focus group.

The focus group was conducted by the primary researcher (facilitator, FY) and supported by a second researcher (moderator, MC). During the focus group, the logic models were presented. A focus group topic guide (Appendix 7) was used to guide discussions whilst allowing flexibility for open feedback.

#### Analysis

The focus group was recorded via MS Teams and transcribed verbatim for analysis. The transcript was analysed alongside the recording to review any discrepancies and used to iteratively refine the logic model and dark logic model. This employed manifest content analysis [28] continuing to use an inductive and deductive coding approach to refine the categories within the logic models. Data was analysed by the primary researcher (FY) and considered any disagreements or deviant case analysis [30]. 

## Results

### Document analysis

Data was extracted from national guidance reports, policies, position statements, rapid case-study evaluations and rapid evidence reviews on virtual wards in the UK, published up to and including March 2024 (Appendix 3). The majority were published between 2022 and 2023. Citation searching revealed further literature, including one randomised-controlled trial [31] and a systematic review that included virtual wards from seven countries [32]. These were included as they contained relevant data on HaH in the UK. The first stage produced the preliminary draft logic models (Appendix 1). Several documents provided information on risks and limitations in virtual wards which helped inform the dark logic model. Whilst undergoing the analysis, it became apparent that the data was aimed at different levels of service delivery, and was logically organised into patient (micro), service (meso) and system (macro) levels, to enable a better understanding of the outcomes and impact in different contexts. Further information to expand on the information in the logic models is also included in Appendix 2.

### Key informant interviews

Twelve participants were interviewed who were professionals involved with virtual wards/ HaH services either from a policy/national guidance level (*n* = 2), service management/administrative (*n* = 1), or service delivery/clinical remit (*n* = 9, of which *n* = 2 were also clinical leads). Key informants represented a wide range of professional backgrounds to provide a comprehensive perspective. These included pharmacists (*n* = 7), consultant geriatrician (*n* = 1), advanced nurse practitioners (*n* = 2), clinical services manager (*n* = 1), and an advanced physiotherapist practitioner (*n* = 1). They covered a variety of specialities (i.e. respiratory, frailty, etc.). Participants were also from a variety of geographical locations; London (*n* = 3), Birmingham (*n* = 3), Newcastle and Northumbria (*n* = 3), Oxford (*n* = 2), Leeds (*n* = 1). Interviews lasted between 30 and 60 min based on participants’ availability.

### Focus group

An online focus group with four participants, a facilitator (FY) and moderator (MC) took place in November 2024 and lasted around 90 min. The participants were experienced health care professionals with current involvement in virtual wards and included three pharmacists with senior roles and a physiotherapist trainee advanced clinical practitioner. Each participant was from a different geographical area in England.

### Key findings

**Fig. 1:**
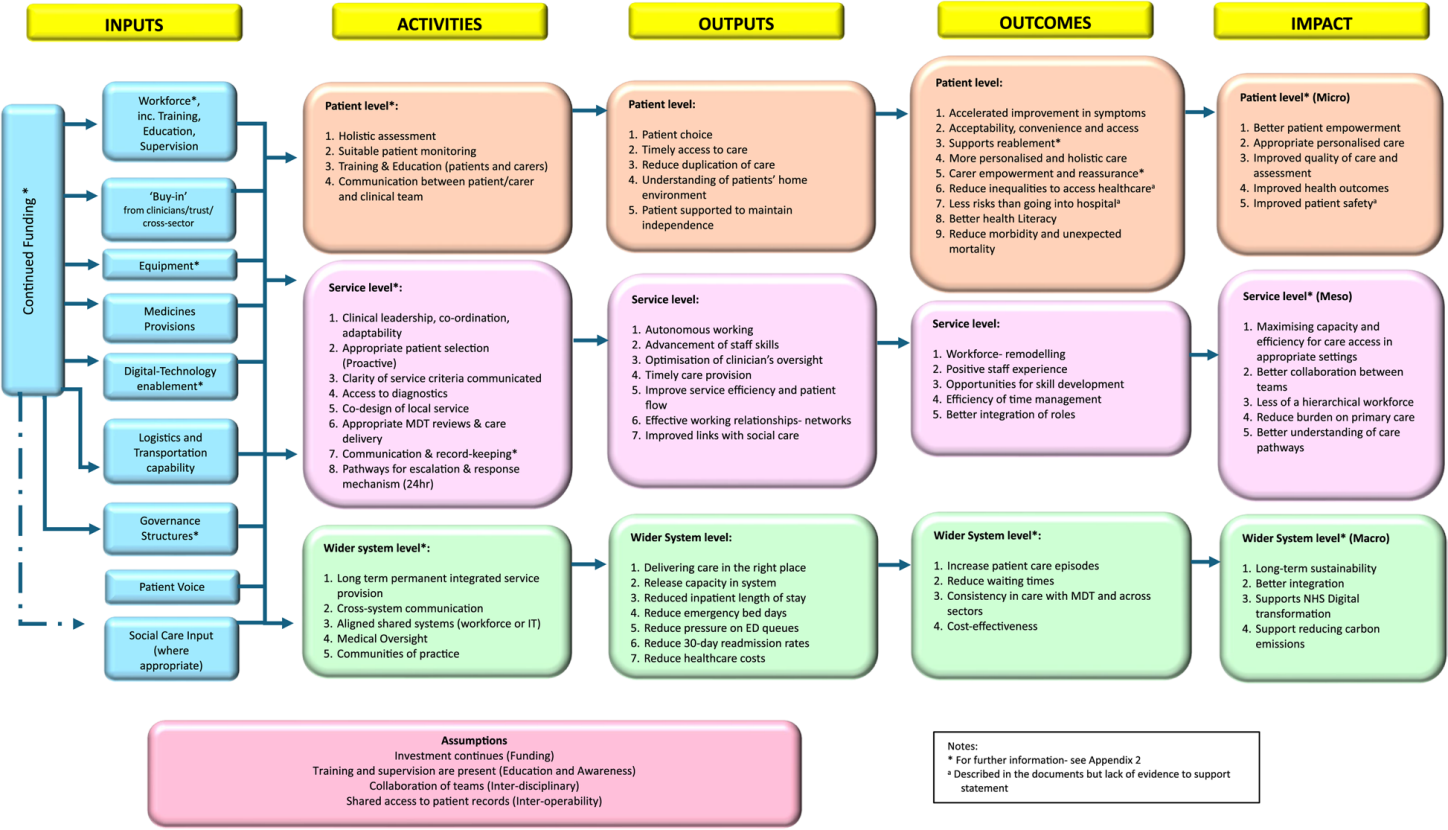
Logic model

**Fig. 2:**
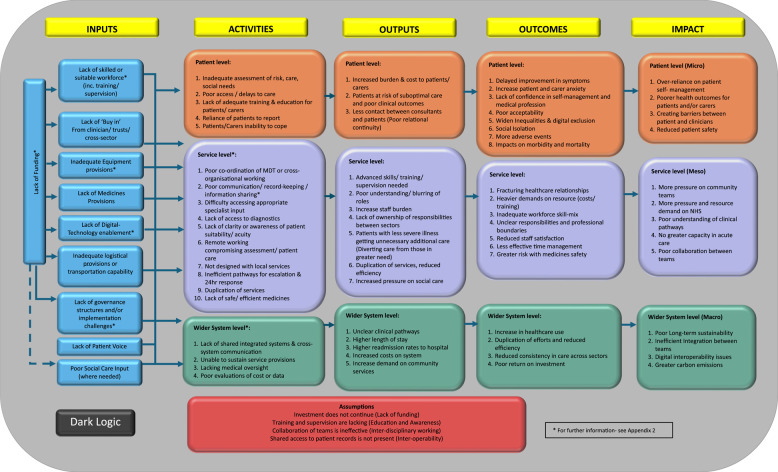
Dark logic model

The data from all workstreams were used to refine the logic models and produce the final versions, displayed in Figs. 1 and 2. The discussions shaped the key components of the logic model. However, key cross-cutting themes were developed as described below:

#### Buy-in

Participants felt to ensure the success and sustainability of the service, securing ‘buy-in’ from all stakeholders (clinicians, primary and secondary care teams, and senior staff) was essential. However, existing hesitancy and lack of awareness have potentially hindered HaH implementation. Without buy-in, there is a risk of fragmented care, barriers to seamless service delivery, and potential duplication of efforts.

*“One of the biggest challenges people have in terms of getting this off the ground is …getting referrals from other clinicians because clinicians don’t understand what (A)- what the concept of a virtual ward is or (B)- what it’s capable of delivering… But there’s just this complete lack of understanding of what a hospital at home service or a virtual ward can deliver*,* and how it delivers it.” (KI06)*

Senior clinical buy-in was essential for the sustainability of HaH models. This was to support clinical decisions and provide oversight for clinical queries. Some participants highlighted current challenges in reaching consultants when they are already responsible for inpatient wards, leading to delays or uncertainty when escalation is needed.

*“…and workforce. I think we’ve seen like the impact if you don’t have like a consultant*,* we’re having to then rely on the consultants in-house…they are then having to again like reach out… to like clinical leads and stuff to kind of support*,* if there are any queries.” (KI07)*

Participants emphasised the importance of incorporating the patient voice from the initial set-up of a HaH service. However, patient engagement was not explicitly accounted for in the current budget, relying on ‘goodwill’ and existing resources. Equally, the involvement of social care partners, local councils, and voluntary organisations (e.g., AgeUK) was viewed as beneficial, depending on the clinical speciality.

#### Holistic personalised care

Participants felt HaH supported patient choice and giving patients autonomy of their care. It supports patients in maintaining independence due to familiarity with their own environment. Equally, the healthcare professionals’ knowledge and awareness of the patients’ environment was found to be a key facilitator in holistic care provision and supporting reablement. For example, understanding how patients will cope after discharge and knowing they will have the correct support in place to manage. Knowledge of both the patients’ home environment and the patients’ condition facilitates the appropriate selection of patients for HaH, as not all patients would be considered suitable. Currently, this relies on clinicians’ judgement as there appears to be limited use of decision support tools to aid assessment of suitability or acuity.

*“There’s lots of patients that we could treat at home*,* but they don’t have an appropriate home environment*,* they need social care set up… And then you’ve got people who are perfectly well set up at home*,* but…their medical condition isn’t conducive to being treated at home…so it is patient specific” (KI06)*

HaH was seen to increase access to care where it’s needed by shifting care to the community, increasing ‘patient care episodes’. “*I don’t know if it’s increasing capacity or if it’s sort of maximising capacity or something to do with the efficiency of it.” (P2)* Participants also described this as increasing access to secondary care beds when appropriate, emphasising “*Delivering… the right type of care to people in the right place” (KI05)*.

Participants also recognised that HaH reduces the risks associated with hospital admissions, and provides personalised care allowing patients to be at home and recover quicker, and reduce morbidity. Discussions suggested this may not necessarily lead to decreased mortality, i.e., if the patient dies in the preferred place of death.

“…*from the… user perspective… I want to be get better quicker and be at home. So you’ve got someone with their acute infection and so they go and be at home*,* they get their intravenous antibiotics*,* but they’re also still getting up*,* moving around and not developing all the dependency of being in hospital.” (KI12)*

#### Workforce re-modelling

“*You’ve got the right person doing the right thing.” (KI01)* Ensuring you have the competent staff to deliver care was important. Most HaH staff are advanced practitioners and prescribers, to enable autonomous clinical decisions to be made, saving the need for multiple clinicians visiting the patient.

*“And you know*,* our MDT will have the nurse*,* the pharmacist and the consultant*,* all of whom are kind of at an equal level…A non-hierarchical model…what we want is people’s maximal empowerment to be an independent clinician” (KI12)*

This creates opportunities and flexibility for skill expansion, but staff need the right training and supervision.

*“We have to know how to go into people’s homes*,* work in the domiciliary setting*,* which is different. You know*,* it’s you’ve got to make sure that you’re safe as well as your patient is” (KI05)*

With the lack of suitable workforce or the training, staff retention and recruitment were voiced, with concerns on service sustainability.

The idea of re-modelling the existing workforce in integrated teams was described by participants to create a new way of efficiently delivering HaH care. “*Thinking [and] working differently*,* where we’re utilising workforce across the system…. outside of …working across traditional organisational boundaries.” (KI03)* This may look like shared workforce across an integrated care system to help strengthen effective working relationships and information sharing. *“I think that shared working systems that could be your workforce*,* that could be your IT” (KI03)* Currently, this relies on established connections and where this is absent, care can seem fragmented. Additionally, challenges over awareness of where HaH service sits, whether that is in primary or secondary care creates practical challenges and having a shared workforce can be a potential solution.

#### Communication

Communication was considered essential between patients, carers and the clinical team, but also within the clinical teams. Giving the patient education on what to do if their condition changes, how to monitor, and key contacts (including out of hours) were important. “*Yeah*,* communication is*,* I would add to that*,* both with the patient*,* the patient’s carers*,* but also the rest of the health system around the patient.” (KI01)*.

Communication at a service level between clinicians and external teams was crucial, including clear communication of service criteria to support awareness and referrals to the service. Participants discussed a potential risk to patients if there were gaps in the communication (such as between district nursing teams, social care, primary care and the HaH staff).

*“Where in hospital they are quite contained because they’re physically on a ward and you can physically keep tabs on them and keep track of them*,* whereas in community*,* you have all these other different services… getting involved and it’s important to have that collaborative approach with them.” (KI04)*

#### Operational capabilities

A suggestion to improve communication was using technology. A common response was the issue with using multiple systems for electronic records and poorly organised, cross-sector working. Duplication in record-keeping was identified as a significant concern requiring improvement. Limited visibility of care information across secondary, primary, and social care sectors created considerable information gaps in the system.

*“So you can’t always see absolutely everything…But if you could see everything*,* then that probably would be more beneficial.” (KI08)*

However, participants recognised that pragmatically this may be difficult to achieve. *“It’s just one of those things that I think’s always putting [in] the too hard to do box” (P1).*

Participants agreed that solutions were required to ensure HaH operates more efficiently, which may include shared systems, dedicated resources for blood tests or transportation, thereby saving a lot of time and reducing risk.

Participants recognised key resources for HaH to operate such as medicines provision and suggested documenting as a separate input. In its absence, operations, governance and safety would be of concern. *“… prescribing a medicine is the most common thing that’s done to treat a patient. It’s the most*,* it’s like the number one thing” (KI05)*.

Furthermore, continued funding was stressed as important to sustain all other required resources “*… it’s funding everything below the resource*,* your equipment*,* your workforce.” (KI03)*, raising concerns about HaH’s sustainability without adequate funding.

### Inequalities

Participants highlighted concerns about the potential for widening inequalities with HaH. These may be due to financial disadvantages for patients, i.e., costs of obtaining prescriptions that would normally be supplied by a hospital. Other inequalities, like digital exclusion or access to rural areas were also highlighted. Participants however acknowledged that HaH improves convenience and access for patients, but agreed evidence is lacking. Discussions around how this outcome is measured was interesting, for example, ‘bed days saved’ versus inclusion.

*“Or is there going to be a pragmatic approach whereby you say*,* well*,* actually I can save 10 hospital beds by treating people close by to us in a high-density population area and the people who are further*,* you know*,* further out that would take the same amount of…?” (P1)*

### Consensus

Overall, consensus was reached on the final logic models, structure and concepts. Participants agreed that the logic and dark logic models were *“useful and very powerful” (P1).* Although a broad understanding of HaH was complex, participants explicitly stated that the level of detail was appropriate for the logic models to be beneficial. It was agreed that the key underlying assumptions underpinning HaH services were namely; continued investment, education and awareness, inter-disciplinary working and inter-operability.

## Discussion

This study explored the complexity of HaH (virtual ward) models in England, looking at both intended and unintended outcomes (logic and dark logic models). It identifies the key ingredients required for successful sustainable HaH models and potential consequences of their absence, based on insights from literature and stakeholders.

Evidence in this field is continually emerging with advancements in practice. Since the initial document analysis was undertaken, the NHSE virtual wards operational framework was published describing ‘what good looks like’ and the core functions and components of virtual wards [33]. Additionally, documents such as ‘Getting it Right First Time’ [34] clearly demonstrate to clinicians what is needed for successful virtual wards in different specialities [34]. However these do not provide the level of detailed insight produced in this studies’ dark logic which helps target quality improvement to the specific areas of need.

This study has identified themes not described in previous literature. Critically, ‘buy-in’ from other clinicians, trusts and sectors was essential for the sustainability of HaH. In one previous study [9] the need of trust and shared goals in a ‘team of teams’ resembling the importance of ‘buy-in’ was highlighted. However, the realities of how this works in practice is not explained by current evidence.

Consistent with the literature, [8, 9, 33, 35–37] this study captured how HaH focuses on personalised and holistic care, as a safe alternative to hospital. However, clear communication and valuing the patient voice is important as an input when setting up HaH services and individual care. This study also stresses the need for clear communication during patient management, including referral criteria, clinical responsibilities and escalation pathways.

Another key theme from this research was the need for improved shared systems in both IT and workforce for more effective working within networks. To foster better working relationships, it was understood that the workforce could be utilised differently. This could involve re-modelling the workforce with shared teams, optimising skills and enhancing access to a range of multi-disciplinary professionals. HaH supports valuable insights into working beyond traditional boundaries and reshaping the hierarchical structures seen in other settings, fostering ambitions for integrated care. This builds on recent evidence that recognises that workforce and technology are key components for functional HaH services [37]. The study by Shi et al. [37] explains that workforce can be either community-based or hospital-based or both, and that technology can be low-intensity (e.g. phone calls) or high-intensity (e.g. wearables). Interviews with stakeholders in our study corroborate these findings taking a practical perspective to consider how this may be applied in a HaH UK setting within integrated care systems.

Participants recognised that current operational capabilities were not adequately designed for seamless delivery of HaH causing practical challenges. These challenges include the need for shared working systems, particularly with digital technology to ensure safe communication. Without this, there is a significant risk of care duplication, resulting in inefficiencies and associated risks. Other operational challenges such as transportation or access to dedicated resources decrease efficiency and effective delivery of HaH. In line with a review by Chen at al [35], medicines/pharmacy provisions were also highlighted as a key resource.

This study complements other evidence syntheses which have also sought to include stakeholder perspectives [10, 35]. Whilst those have adopted a realist review approach developing programme theory in different contexts, the simplified but granular findings of this study, especially in the dark logic will be useful for practical application in all HaH contexts.

### Strengths and limitations

To our knowledge, this is the first piece of work that investigates patient risk within HaH using a dark logic model. Whilst some logic models have been developed based on individual service evaluations [38], this is the first piece of work that develops logic models using a combination of national documents, policies, current literature in England, validated with stakeholder involvement. Furthermore, triangulation of methods and the wide geographical remit of this work makes it robust and generalisable to inform future HaH program planning and implementation.

Although not a systematic review with critical appraisal, the literature has been reviewed within the document analysis in a systematic manner guided by relevant methodological approaches in the literature. This work is innovative as there are very limited dark logic models published, taking a contemporary angle to evaluate risk and research priority setting. The authors acknowledge that pharmacists were slightly over-represented in the sample due to the authors networks for recruitment and the likely changing composition of HaH teams in the UK adopting advanced pharmacist roles. Furthermore, the logic models were intended to be simple illustrations for two reasons. (1) They can be easily visualised by busy practitioners and program managers who may find the logic models useful for planning, implementation or evaluation. (2) They align with the philosophy described for developing logic models - ‘if’, ‘then’, ‘so that’ statements [11]. 

### Gaps in evidence and further questions

**Fig. 3:**
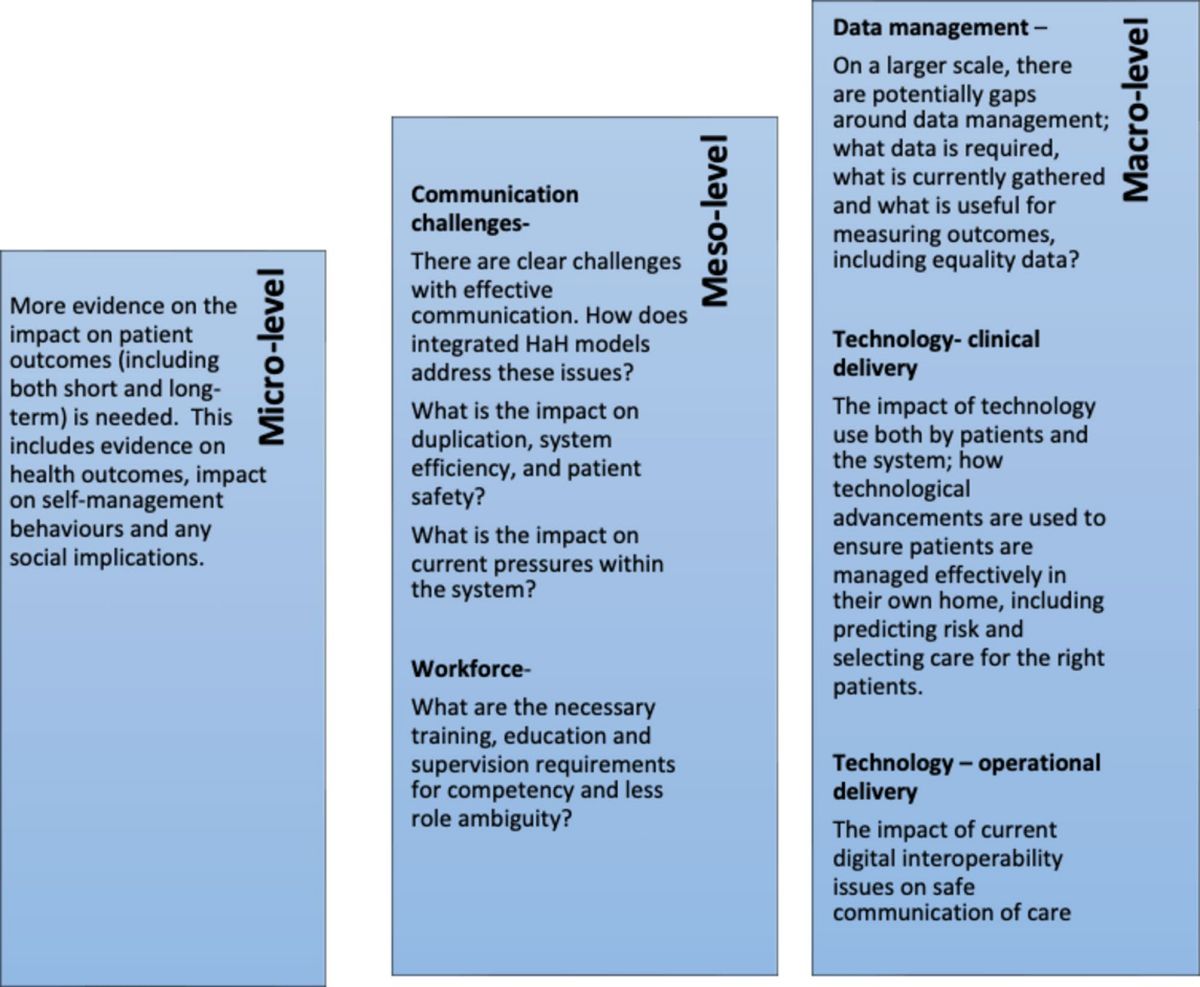
Evidence gaps and potential considerations for research

As a result of this work, the dark logic model has enabled us to understand potential risks, challenges and research questions that are yet to be answered, from various angles (See Fig. 3).

### Implications for practice and further work

These logic models serve as robust evidence-based visual tools to guide healthcare systems in exploring implementation or evaluation strategies. Invaluably, the dark logic model allows us to visualise hypothetical unintended consequences and target areas of concern to improve services. This supports policymakers to consider gaps in the system and how we should prioritise future research and evaluation to ensure service sustainability, patient safety and cost-effectiveness. It is important to recognise that logic models are dynamic and can be adaptive with time, context and advancement in services. Therefore, will need updating with emerging research or changes in practice.

For those seeking to understand the deeper ‘why’, ‘what’ and for ‘whom’, the logic models can form the basis towards developing program theory as supported by the Medical Research Council framework for developing and evaluating complex interventions [39]. Other methods to understand causal pathways and mechanisms of change, for implementation strategies, exploring mediators, moderators and preconditions may be useful such as adopting the four-step approach as described by Lewis et al. [40] to build on these logic models. Alternatively, to explore change concepts, breaking down the individual aspects and developing driver diagrams may be helpful for quality improvement [41]. 

This will support identification of what changes are needed and ‘how’ we can achieve these. For example, continuous funding was emphasised by most participants as a limiting factor, and it would be useful to consider what needs to happen as a precursor to support this. It may be useful for policy makers or integrated care system leaders to explore this further by optimising and adapting the logic models in local context. Moreover, considering the patient view would be a useful perspective and likely yield different findings with regards to hidden or unintended consequences on the dark logic model.

## Conclusion

This study provides a visual overview of the intended outcomes (logic model) and potential unforeseen consequences (dark logic model) of hospital at home (virtual wards) in England. It breaks down the key components and provides a visual map to establish areas for program planning or seeking to improve quality of care and safety. The key themes for sustainability were having ‘buy-in’, effective communication, ensuring operational capabilities were optimised as well as utilising the workforce differently. Future research should seek to explore strategies to measure safety and how we can maximise the potential of current resources, including digital technology to improve care communication and integration. Incorporating the patient and carer perspective and the impacts on equity would also be useful.

## Supplementary Information

Supplementary Material 1: Appendix 1. Draft Logic Models from Document Analysis.

Supplementary Material 2: Appendix 2. Additional Information from Document Analysis to supplement draft logic models.

Supplementary Material 3: Appendix 3. Document Analysis table.

Supplementary Material 4: Appendix 4. Phase 2 Logic Models after refinement through Key Informant Interviews.

Supplementary Material 5: Appendix 5. Focus group- Summary of data.

Supplementary Material 6: Appendix 6. Semi-structured Interview guide.

Supplementary Material 7: Appendix 7. Focus Group topic guide.

## Data Availability

Anonymised data used and/or analysed during the current study are available upon reasonable request from the corresponding author.

## Abbreviations

HaH: Hospital at Home
MDT: Multi–disciplinary team
NHS: National Health Service
NHSE: National Health Service England
UK: United Kingdom
