# Supplementary figures and images for: Hospital at home (virtual wards): developing a logic model and dark logic model
Source: BMC Health Serv Res. 2025 May 17;25:714. doi: 10.1186/s12913-025-12872-w (PMC12085072; doi:10.1186/s12913-025-12872-w)

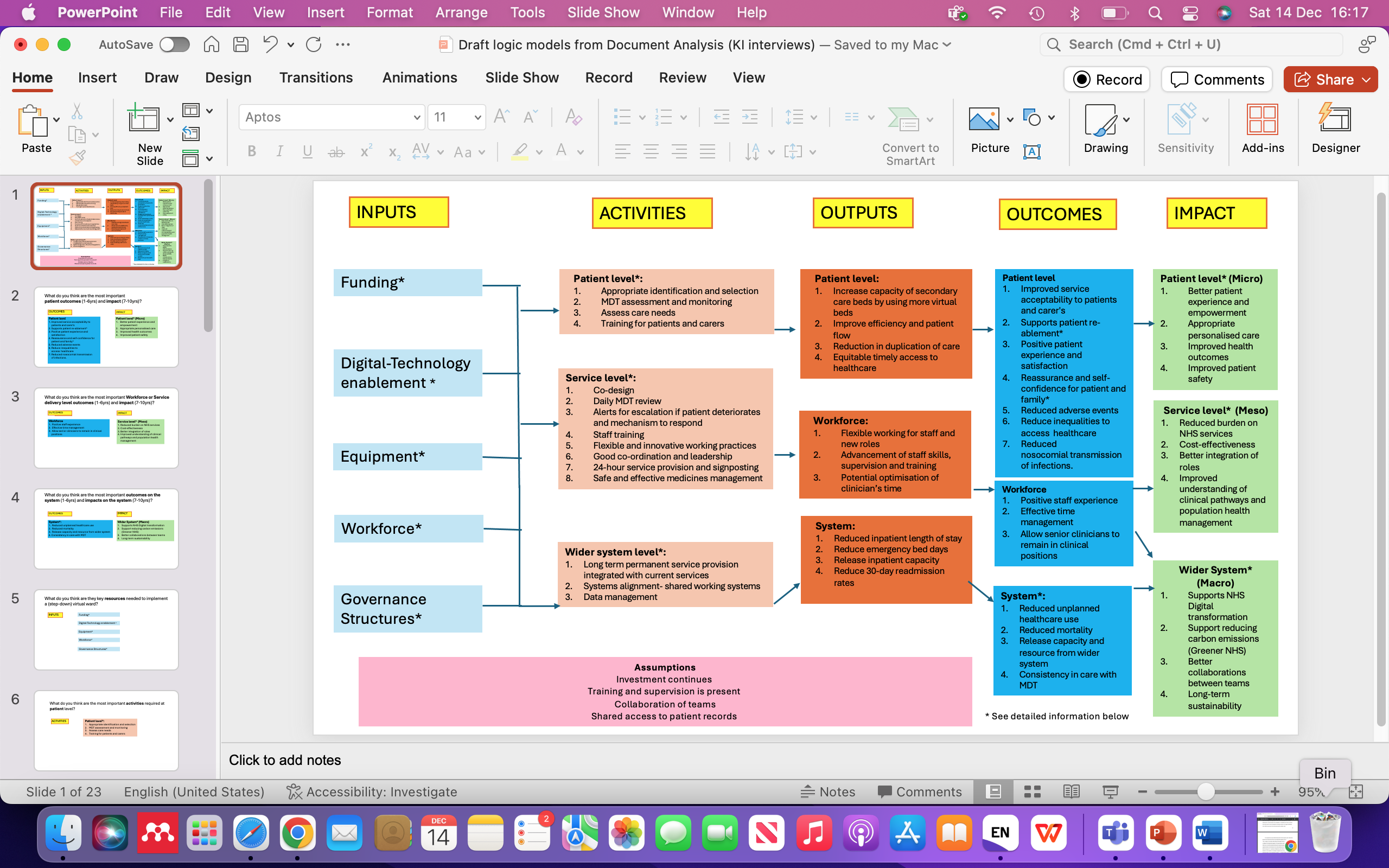


**Appendix 1. Draft Preliminary Logic model and Dark logic model**


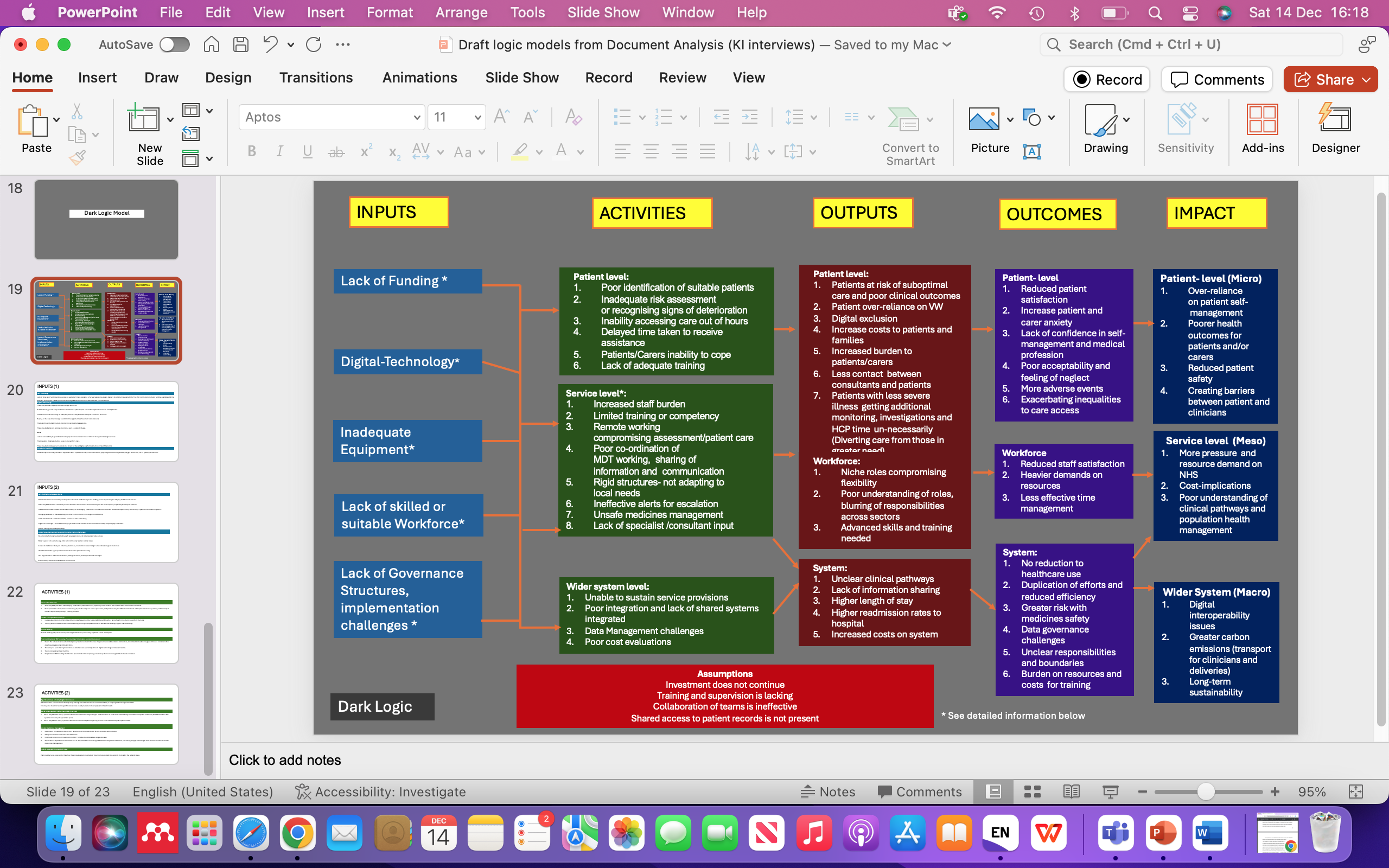

Supplement: Supplementary file 1 — Supplementary Material 1: Appendix 1. Draft Logic Models from Document Analysis. [file 12913_2025_12872_MOESM1_ESM.docx]
