# Supplementary material for: Hospital at home (virtual wards): developing a logic model and dark logic model
Source: BMC Health Serv Res. 2025 May 17;25:714. doi: 10.1186/s12913-025-12872-w (PMC12085072; doi:10.1186/s12913-025-12872-w)
Supplement: Supplementary file 2 — Supplementary Material 2: Appendix 2. Additional Information from Document Analysis to supplement draft logic models. [file 12913_2025_12872_MOESM2_ESM.docx]

# Appendix 2: Additional Information from Document Analysis to supplement draft logic models.

# Logic Model

## INPUTS


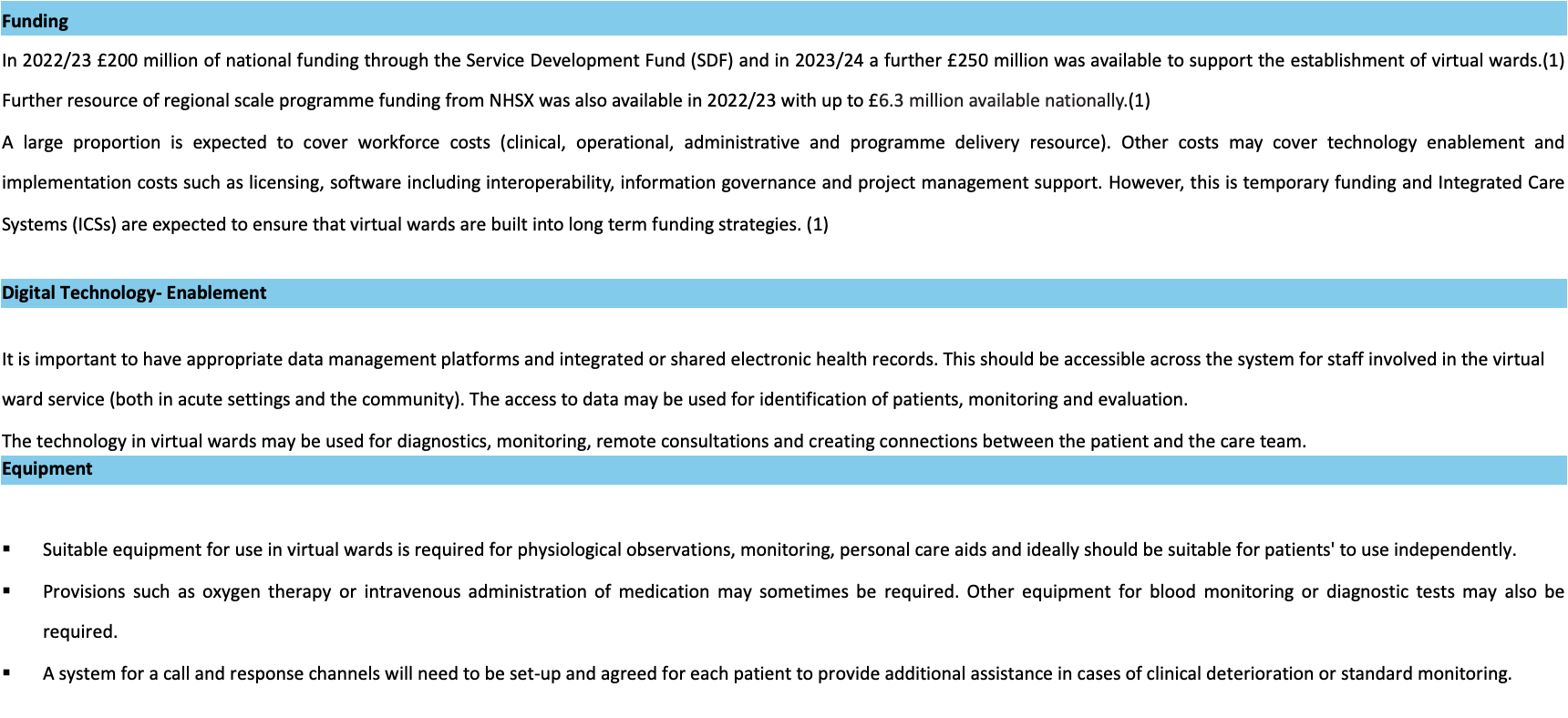


References: 1. NHS. Supporting information for ICS leads Enablers for success: virtual wards including hospital at home. National Health Service; 2022.

| Workforce |
| --- |
| A workforce consisting of a multi-disciplinary team (MDT), including links between secondary care, primary care, emergency services, and community teams including social care.It should be led by a named consultant practitioner (including a nurse or AHP consultant) or suitably trained GP with relevant experience and training, with clear lines of clinical responsibility and governance.It should have a suitable skill-mix to provide leadership, full accountability, risk stratification, education and training and include specialist nurses.A virtual ward coordinator should also be responsible for good coordination of the team, workflow and evaluation.Training structures such as a workforce learning and development strategy for providing acute care at home, should be in place. |
| Governance structures |
| These should include:Common standards agreements and SOPs (i.e. for patient eligibility, assessment procedures, care documentation, data protection, safeguarding, and discharge.)Clear defined criteria should be in place to cover triage/ risk assessment/ inclusion/ referral /escalation and clear agreed pathways for care.Data management agreements for training, procurement, and information governance (I.e. a data processing impact assessment (DPIA), a data sharing agreement (DSA), a data processing agreement (DPA),  a privacy statement.)Systems for reporting and evaluationLogistical structures for deliveries of equipment and/or medication.Clear service provisions for out of hours and 24/7 access to care |

## Activities

| **Patient-level** |
| --- |
| Identification of suitable patients - this can be achieved through a clinical coordinator (advanced nurse practitioner, GP, consultant) or with the support of data or risk scores. |
| Managing risk appropriately- risk assessments and procedures, to take place in patients' homes. Obtaining patient consent and agreement |
| Monitoring and multiprofessional clinical assessments -(including proactive monitoring), Ensure monitoring is tailored to the patients' needs. |
| Supporting personal care needs |
| Training for patients and carers on monitoring and ensuring they know when to seek support - Ensure patients feel supported and confident in self-management and monitoring |
| **Service level** |
| Co-design of service and interventions (understanding local needs and co-production with patients) |
| Daily MDT meetings - daily board rounds or huddles to review patients. These can be prioritised by the level of care needed, risk scores, or traffic light coding (red, amber, green) |
| Alerts for escalation if patient deteriorates and how to respond or refer |
| Staff Training, with accountability and supervision |
| Multi-agency approach, including speciality assessments, partnerships and collaborations with existing teams, good relationships between services |
| Working across boundaries; Workforce flexibility in roles (hybrid roles), working patterns, remote working. |
| Good co-ordination and leadership |
| 24hour service provision and signposting |
| Safe and effective medicines management- Timely access to medicines and care, medication reconciliation, medication review, administration systems, reducing polypharmacy, antimicrobial stewardship) |
| **Wider System** |
| Long-term permanent service provision integrated with current services |
| Systems alignment is needed. Having shared working systems; enabling remote monitoring, video conferencing, F2F or telephone. Clear knowledge and information sharing needed. |
| Data management: Data protection, integration and better use of data. Using shared data can also aim to reach people who are at higher risk of inequalities.  Clear documentation and care planning  is also needed. |

## **Outcomes**

| **Patient Specific** |
| --- |
| **Patient reablement**  1. Prevents patients getting deconditioned and experiencing functional decline 2. Increased patient mobility  3. Reduced need for community support after discharge 4. Reduced admission to residential care |
| **Reassurance and self-confidence for patient and family** |
| 1. Increased family's peace of mind and reduce anxiety 2. Patients feeling better prepared for transitions   **Reduced Adverse Events** |
| Reduce risk of being prescribed >2 medicines that can result in acute kidney injury (AKI) |
|  |
| **System** |
| Reduced use of Emergency department (ED)/ hospital attendances and unplanned contacts/treatment events. |
| Reduced mortality by improving timely access to care in patient's homes, supporting recovery and improving health outcomes |
| Reduce elective waiting times and a reduction in use of outpatient services. |
| Encourages discussions between care professionals and patients and enables a greater clinician and patient understanding |
|  |

## **Impact**

| **Patient** |
| --- |
| Better patient experience and Empowerment |
| Increased patient choice and satisfaction |
| Increased acceptability and improved patient experience |
| Less Disruption to people's lives |
| Better patient self-management and confidence |
| An empowered culture |
| Appropriate Personalised care |
| Personalised care – the right patient is treated with the right skills and is given the right care at the right time |
| Improved health outcomes |
| Improved patient safety |
| Improved medication management and safety |
|  |
| **Care System** |
| Reduced burden on NHS services by keeping people at home (reduces the burden on the healthcare system allowing capacity for other services) |
| Reduced Unplanned healthcare use, increases bed capacity |
| Cost effectiveness -Cost-savings and positive return-on-investment,  Better Value services for the NHS |
| Better Integration of roles |
| Improved understanding of clinical pathways and population health management |
| Improved understanding of clinical pathways |
| Better understanding of patient demographics /local needs |
|  |
| **Wider system** |
| Supports NHS digital transformation |
| Support NHS Digital transformation and use of digital technologies across systems  Facilities for automated data collection  Better sharing of information |
| Support reducing carbon emissions (Greener NHS) |
| Better Collaborations between teams |
| Long-term sustainability |
| Part of long-term strategy |
| Financially stable and sustainable |

# Dark Logic

## **INPUTS**

| Lack of Funding |
| --- |
| Lack of long-term funding and resources to sustain efficient operation of virtual wards may create barriers to long-term sustainability. The short-term and structured funding available and the delays in funding can create operational challenges and barriers to the effectiveness of virtual wards. |
| Digital-Technology |
| There may be lack of appropriate technology resources.  If the technology is not easy to use for both staff and patients, this can create digital exclusion for some patients.  The use of remote monitoring for older people with frailty and other complex conditions is limited.  Relying on the use of technology could limit the opportunities for patient consultations  The lack of trust in digital remote monitoring can lead to false alarms.  There may be barriers to remote  monitoring such as patient refusal.  Data:  Lack of accessibility to good data on local population needs can make it difficult to target and design services .  The relaxation of data protection rules comes with it’s risks.  There may be multiple patient records due to lack of shared digital platforms (Electronic Health Records). |
| Inadequate Equipment |
| Patients may need timely access to equipment such as personal aids, incontinence aids, physiological monitoring devices, oxygen which may not be speedily accessible. |

| Lack of Funding |
| --- |
| Lack of long-term funding and resources to sustain efficient operation of virtual wards may create barriers to long-term sustainability. The short-term and structured funding available and the delays in funding can create operational challenges and barriers to the effectiveness of virtual wards. |
| Digital-Technology |
| There may be lack of appropriate technology resources.  If the technology is not easy to use for both staff and patients, this can create digital exclusion for some patients.  The use of remote monitoring for older people with frailty and other complex conditions is limited.  Relying on the use of technology could limit the opportunities for patient consultations  The lack of trust in digital remote monitoring can lead to false alarms.  There may be barriers to remote  monitoring such as patient refusal.  Data:  Lack of accessibility to good data on local population needs can make it difficult to target and design services .  The relaxation of data protection rules comes with it’s risks.  There may be multiple patient records due to lack of shared digital platforms (Electronic Health Records). |
| Inadequate Equipment |
| Patients may need timely access to equipment such as personal aids, incontinence aids, physiological monitoring devices, oxygen which may not be speedily accessible. |

## **ACTIVITIES**

| Increased staff burden |
| --- |
| 1. Staff may be expected to travel varying distances to patient's homes, especially if not close to the hospital/base and rural environments. 2. Work patterns are likely to be varied or long to provide adequate service provisions, shift patterns may be different to their role in hospital or community setting and flexibility in hours is expected especially if needing to travel. |
| Limited training and competency |
| 1. Inadequate and unclear training and training pathways may blur responsibilities and lead to a lack of staff competency to perform their role. 2. Training and considerations for remote working, working in people's homes as well as lone working support may be lacking. |
| Remote working |
| Remote working may result in compromising assessments, monitoring or patient care if inadequate. |
| Poor coordination of MDT working, Poor sharing of information and communication |
| 1. Due to the nature of various multidisciplinary teams involved in the care of a patient across boundaries and sectors, consideration needs to be given in how to coordinate this care to avoid gaps or avoid duplication 2. There may be poor sharing information or data between systems (both with Digital technology or between teams) 3. Teams not working in partnership 4. Disparities in MDT meeting attendances (due to lack of time/capacity) could delay-decision making and demotivate attendees |

| Increased staff burden |
| --- |
| 1. Staff may be expected to travel varying distances to patient's homes, especially if not close to the hospital/base and rural environments. 2. Work patterns are likely to be varied or long to provide adequate service provisions, shift patterns may be different to their role in hospital or community setting and flexibility in hours is expected especially if needing to travel. |
| Limited training and competency |
| 1. Inadequate and unclear training and training pathways may blur responsibilities and lead to a lack of staff competency to perform their role. 2. Training and considerations for remote working, working in people's homes as well as lone working support may be lacking. |
| Remote working |
| Remote working may result in compromising assessments, monitoring or patient care if inadequate. |
| Poor coordination of MDT working, Poor sharing of information and communication |
| 1. Due to the nature of various multidisciplinary teams involved in the care of a patient across boundaries and sectors, consideration needs to be given in how to coordinate this care to avoid gaps or avoid duplication 2. There may be poor sharing information or data between systems (both with Digital technology or between teams) 3. Teams not working in partnership 4. Disparities in MDT meeting attendances (due to lack of time/capacity) could delay-decision making and demotivate attendees |

## References:

1. NHS. Supporting information for ICS leads Enablers for success: virtual wards including hospital at home. National Health Service; 2022.
