# Supplementary material for: Hospital at home (virtual wards): developing a logic model and dark logic model
Source: BMC Health Serv Res. 2025 May 17;25:714. doi: 10.1186/s12913-025-12872-w (PMC12085072; doi:10.1186/s12913-025-12872-w)
Supplement: Supplementary file 4 — Supplementary Material 4: Appendix 4. Phase 2 Logic Models after refinement through Key Informant Interviews. [file 12913_2025_12872_MOESM4_ESM.docx]

INPUTS

ACTIVITIES

OUTPUTS

OUTCOMES

IMPACT

**Patient level*:**

1. Holistic assessment
2. Suitable patient monitoring
3. Training & Education (patients and carers)
4. Communication between patient/carer and clinical team

**Patient level:**

1. Patient preference
2. Timely access to care
3. Reduce duplication of care
4. Understanding of patients’ home environment
5. Patient supported to maintain independence

**Patient level:**

1. Accelerated improvement in symptoms
2. Acceptability, convenience and Access
3. Supports reablement*
4. More personalised and holistic care
5. Carer empowerment and reassurance*
6. Reduce inequalities to access healthcare^a^
7. Less risks than going into hospital^a^
8. Better health Literacy
9. Reduce morbidity and unexpected mortality

Funding *

Workforce*,

inc. Training, Education, Supervision

**Patient level* (Micro)**

1. Better patient empowerment
2. Appropriate personalised care
3. Improved quality of care and assessment
4. Improved health outcomes
5. Improved patient safety^a^

‘Buy-in’

from clinicians/trust/ cross-sector

**Service level*:**

1. Clinical leadership, co-ordination, adaptability
2. Appropriate patient selection (Proactive)
3. Communication of service criteria
4. Access to diagnostics
5. Co-design of local service
6. Appropriate MDT reviews & care delivery
7. Communication & record-keeping*
8. Pathways for escalation & response mechanism (24hr)
9. Safe, effective medicines management & provision
10. Transportation

Equipment*

**Service level* (Meso)**

1. Increase capacity in acute settings
2. Better collaboration between teams
3. Less of a hierarchical workforce
4. Reduce burden on primary care
5. Better understanding of care pathways

Medicines Provisions

**Service level:**

1. Autonomous working
2. Advancement of staff skills
3. Optimisation of clinician’s oversight.
4. Timely provision to care
5. Improve service efficiency and patient flow
6. Effective working relationships- networks
7. Improved links with social care

Digital-Technology enablement*

**Service level:**

1. Positive staff experience
2. Efficiency of time management
3. Workforce- remodelling
4. Opportunities for skill development
5. Better integration of roles

Logistics and Transportation capability

**Wider System* (Macro)**

1. Long-term sustainability
2. Better integration
3. Supports NHS Digital transformation
4. Support reducing carbon emissions

**System:**

1. Delivering care in the right place
2. Release capacity in system
3. Reduced inpatient length of stay
4. Reduce emergency bed days
5. Reduce pressure on ED queues
6. Reduce 30-day readmission rates
7. Reduce healthcare costs

Governance Structures*

**Wider system level*:**

1. Long term permanent integrated service provision
2. Cross-system communication
3. Aligned shared systems (workforce or IT)
4. Communities of practice
5. Medical Oversight

**System*:**

1. Increase patient care episodes
2. Reduce waiting times
3. Consistency in care with MDT and across sectors
4. Cost-effectiveness

**Assumptions**

Investment continues (Funding)

Training and supervision are present (Education and Awareness)

Collaboration of teams (Inter-disciplinary)

Shared access to patient records (Inter-operability)

Notes:

* See detailed information in narrative below

^a^ Described in the documents but lack of evidence to support statement

Amendments from Phase 1

New terms – red

Moved terms- Blue

**Patient- level (Micro)**

1. Over-reliance on patient self- management
2. Poorer health outcomes for patients and/or carers
3. Creating barriers between patient and clinicians
4. Reduced patient safety

**Patient- level:**

1. Delayed improvement in symptoms
2. Increase patient and carer anxiety
3. Lack of confidence in self-management and medical profession
4. Poor acceptability
5. Widen Inequalities & digital exclusion
6. Social Isolation
7. More adverse events
8. Impacts on morbidity and mortality

**Patient level:**

1. Inadequate assessment of risk/ care/ social needs
2. Poor access/ delays to care
3. Lack of adequate training & education for patients/carers
4. Reliance of patients to report
5. Patients/Carers inability to cope

**Patient level:**

1. Increased burden &cost to patients/ carers
2. Patients at risk of suboptimal care and poor clinical outcomes
3. Less contact between consultants and patients (Poor relational continuity)
4. Patient over-reliance on service

Lack of skilled or suitable workforce*

(inc. training/ supervision)

OUTPUTS

OUTCOMES

ACTIVITIES

INPUTS

IMPACT

Lack of Funding*

Lack of ‘Buy in’

From clinician/ trusts/ cross-sector

**Service level*:**

1. Poor co-ordination of MDT working
2. Difficulty accessing appropriate specialist input
3. Lack of access to diagnostics
4. Lack of communication of patient suitability/ acuity
5. Remote working compromising assessment/ patient care
6. Poor communication/ record-keeping / information sharing*
7. Not designed with local services
8. Inefficient pathways for escalation & 24hr response
9. Duplication of services
10. Lack of safe/efficient medicines management provisions
11. Challenges with transportation

**Service:**

1. Advanced skills/training/ supervision needed
2. Poor understanding/ blurring of roles
3. Increase staff burden
4. Lack of ownership of responsibilities between sectors
5. Patients with less severe illness getting unnecessary additional care (Diverting care from those in greater need)
6. Increased pressure on social care
7. Duplication of services, reduced efficiency

Inadequate Equipment provisions*

**Service level (Meso)**

1. More pressure on community teams
2. More pressure and resource demand on NHS
3. Poor understanding of clinical pathways
4. No greater capacity in acute care
5. Poor collaboration between teams

**Service:**

1. Fracturing healthcare relationships
2. Heavier demands on resource (costs/ training)
3. Inadequate workforce skill-mix
4. Unclear responsibilities and professional boundaries
5. Reduced staff satisfaction
6. Less effective time management
7. Greater risk with medicines safety

Lack of Medicines Provisions

* See detailed information below

Dark Logic

**Assumptions**

Investment does not continue

Training and supervision are lacking (Education and Awareness)

Collaboration of teams is ineffective (Inter-disciplinary working)

Shared access to patient records is not present (Interoperability)

Amendments from Phase 1

New terms – red

Moved terms- Blue

**Wider System (Macro)**

1. Poor Long-term sustainability
2. Inefficient Integration between teams
3. Digital interoperability issues
4. Greater carbon emissions

**System:**

1. Increase in healthcare use
2. Duplication of efforts and reduced efficiency
3. Reduced consistency in care across sectors
4. Poor return on investment

**Wider system level*:**

1. Lack of shared integrated systems & cross-system communication
2. Unable to sustain service provisions
3. Lacking medical oversight
4. Poor evaluations of cost or data

**System:**

1. Unclear clinical pathways
2. Higher length of stay
3. Higher readmission rates to hospital
4. Increased costs on system
5. Increase demand on community services

Lack of Digital-Technology enablement*

Inadequate logistical provisions or transportation capability

Lack of governance structures and/or implementation challenges*
