# Supplementary material for: Hospital at home (virtual wards): developing a logic model and dark logic model
Source: BMC Health Serv Res. 2025 May 17;25:714. doi: 10.1186/s12913-025-12872-w (PMC12085072; doi:10.1186/s12913-025-12872-w)
Supplement: Supplementary file 6 — Supplementary Material 6: Appendix 6. Semi-structured Interview guide. [file 12913_2025_12872_MOESM6_ESM.docx]

# Appendix 6. Semi-structured Interview Topic Guide

**Aim:**

Our aim is to explore current strategies and components involved in ‘step-down’ virtual wards, exploring resources, activities, outputs, outcomes, and impact. This will inform the development of a logic model that can be used for future research and program planning.

**Prior to starting:**

- Thank you so much for making the time to talk to me.
- You have been sent an information sheet and consent form which you have read and completed. Can we confirm that you still consent to participate?
- Today we invite you for a discussion around ‘step-down’ virtual wards. We have looked at the literature available on virtual wards, but we would like to hear your perspective on strategies, risks and components around virtual wards.
- We will give a short presentation on the analysis of the documents and an idea on drafted virtual ward logic models. We invite you to discuss your thoughts on this, if you think it is an accurate reflection and what you may change or add to these components. We may also discuss your thoughts on the links between the components.
- I will be recording the interview to focus on what you are saying without the need to write down lots of notes and distract you.
- It is important to know that there are no right and wrong answers, just be yourself and answer the questions honestly.
- All the discussion in this meeting will be treated confidentially, your responses will be stored in an anonymous format, and so your names will not appear in any report.
- Have you got any questions before we begin recording?

**During Interview:**

- Present information
- Allow participant to share their views.
- Focus the discussion on each component of the logic model: resources, activities, outputs, outcomes, and impact. (Starting with intended results: Impact and Outcomes then resources, activities, and outputs)
- Complete the interview with discussing any links between components.
- If timing allows, discuss visual representation of Logic models
- Finish by thanking participant for their time and contribution.
