# Supplementary material for: Hospital at home (virtual wards): developing a logic model and dark logic model
Source: BMC Health Serv Res. 2025 May 17;25:714. doi: 10.1186/s12913-025-12872-w (PMC12085072; doi:10.1186/s12913-025-12872-w)
Supplement: Supplementary file 7 — Supplementary Material 7: Appendix 7. Focus Group topic guide. [file 12913_2025_12872_MOESM7_ESM.docx]

# Appendix 7. Stakeholder Focus Group Topic Guide

**Aim:**

Our aim is to refine and develop logic model(s) and dark logic models that have been developed through document analysis and key informant interviews.

**Prior to starting:**

- Thank you so much for making the time to talk to me.
- Have you had a chance to read through the information sheet that was sent out to you?
- Today we invite you for a discussion around ‘step-down’ virtual wards. We have drafted and revised proposed logic models based on previous document analysis and key informant interviews.
- Today we present a draft and would like your feedback and input on refining these.
- I will be recording the interview to focus on what you are saying without the need to write down lots of notes and distract you.
- It is important to know that there are no right and wrong answers, just be yourself and answer the questions honestly.
- All the discussion in this meeting will be treated confidentially, your responses will be stored in an anonymous format, and so your names will not appear in any report.
